# The Flammability and Thermal Stability of Filling Epoxy Foam Plastics for Construction Purposes

**DOI:** 10.3390/ma17215268

**Published:** 2024-10-29

**Authors:** Svetlana Samchenko, Maxim Ushkov, Vladimir Erofeev, Valentin Ushkov, Irina Stepina

**Affiliations:** 1 Department of Building Materials Science, National Research Moscow State University of Civil Engineering, Yaroslavskoe sh. 26, 129337 Moscow, Russia; samchenkosv@mgsu.ru (S.S.); erofeevvt@bk.ru (V.E.); va.ushkov@yandex.ru (V.U.); 2 Research Laboratory Modern Composite Building Materials, National Research Moscow State University of Civil Engineering, Yaroslavskoe sh. 26, 129337 Moscow, Russia; ushkovmv@mgsu.ru

**Keywords:** polyepoxide foams, oxygen index, apparent density, flame spreading speed, heat resistance, modifiers, plasticizers, flame retardants, strength, combustibility

## Abstract

An effective type of polymer heat-insulating material (foams) based on reactive oligomers is casting epoxy foams with high technological and operational parameters. However, polyepoxide foams are highly flammable, which significantly restrains their application in the construction industry. The aim of this work was to develop effective methods for reducing the flammability of filling epoxy foams. In order to achieve the objective, the following objectives were addressed: determining the influence of the chemical nature and content of additive and reactive bromine- and phosphorus-containing compounds on the thermal stability, flammability and operational properties of filling epoxy foams, and the development of polyepoxy foams of reduced flammability with high-quality physical and mechanical characteristics. When estimating the flammability of epoxy foams, we used both state-approved methods and the methods described in scientific and technical literature. The thermal properties of epoxy foams were studied with the help of multimodular thermoanalytical complex DuPont-9900. The data on the influence of the apparent density of foams and oxygen concentration in the oxidant flow on the flame propagation speed on the horizontal surface of polyepoxy foams are presented. It was revealed that the chemical nature of amine hardeners does not affect the thermal stability and flammability of epoxy foams. It was established that phosphate plasticizers are ineffective flame retardants of foamed epoxy resin, and the chemical structure of additive organobromic flame retardants insignificantly affects their efficiency. It was shown that microencapsulated flame retardants are inferior in flame retardant efficiency to additive flame retardants. It was found that effective flame retardants for casting polyepoxy foams are phosphorus-containing oligoether methacrylate and epoxidized waste from the production of tetrabromodiphenylpropane. The results of this research will form the basis for the production of an experimental industrial batch of samples of pouring epoxy foams of reduced flammability.

## 1. Introduction

The introduction of strict standards for the thermal protection of buildings and structures, and thermal insulation of technological equipment, regulated, respectively, by SP 50.13330.2012 and SP 61.13330.2012, has led to the use of effective thermal insulation materials in the construction industry [1]. Modern effective thermal insulation materials are poro and foam plastics based on thermoplastic and thermosetting polymers, which have a low apparent density (15–200 kg/m^3^) and thermal conductivity (0.022–0.055 (W/(m∙K)), wide temperature range of applications (from −180 to +150 °C), and good combination of strength and density [2,3,4,5,6,7]. Polymer thermal insulation materials (PTMs) increase the reliability and durability of building structures, reduce heat losses, increase productivity and improve working conditions during construction and installation works. The required range of PTM densities is determined by the field of their application.

Foam plastics can be made practically from any polymer, the choice of which is determined by the required technological and operational characteristics of PTMs, as well as the cost effectiveness of the production process of heat-insulating products on their basis. The performance properties of foams depend on the chemical nature of the polymer matrix and dispersed phase, porosity, pore size and shape, and the production technology of thermal insulation products [2,3,4,7]. At the same time, foams based on reactive oligomers have higher strength, heat and chemical resistance compared to gas-filled thermoplastics.

Foams based on reactive oligomers also include polyepoxy foams (foam epoxyplast, epoxy foams and poroplasts), which are produced on the basis of epoxy and epoxy novolac resins with the addition of volatile hydrocarbons as foaming agents, and di- and polyamines as hardeners [4,5,7]. To increase the heat resistance of polyepoxide foams (PEFs), aromatic polyamines (phenylenediamine, diaminodiphenylmethane) are used as hardeners, and polyamine carbamates, azo compounds, hydrazides and borohybrides are used as gas formers. To reduce the density of PEFs, boron fluoride complexes with alcohols, esters or amines are used as hardeners, and refrigerants (freons) are used as foaming agents. When surfactants are introduced, the foaming of polyepoxy foams occurs at room temperature and curing takes several minutes. Most polyepoxy foams are rigid foams with a closed cell structure [4,5,7].

The advantages of PEFs are high strength, heat and sound insulation properties, dielectric characteristics, heat resistance and a wide range of operating temperatures (−50 to +150 °C), chemical resistance, low water absorption (except for open porous epoxy foams) and high adhesion to various substrates. PEFs in the construction industry are used as heat- and sound-insulating materials in the production of sandwich panels and roofing insulation boards. They are used as electrical insulating material in radio engineering, and in the production of structural products in aviation, automotive, shipbuilding and instrument-making industries. The performance properties and the main methods of modification and reinforcement of epoxy polymers and composites based on them are considered in the works of some scientists [8,9,10].

In Russia, two main methods of polyepoxy foam production have been developed: PE-grade (foaming of foams occurs at elevated temperatures) and PEF-grade (foaming at 20–25 °C). The epoxy foams of PE-1, PE-2, PE-2T, PE-3 and PE-5 grades are obtained by the foaming and curing of ED-16 epoxy resin with amine hardeners. Foams of PE-1, PE-2, PE-2T and PE-3 grades are foamed with the help of ChKhZ-57 porophore, and PE-5 PPE is foamed with the help of Hladon-113 (manual pouring) or Hladon-142 (machine pouring). PE-6 foam plastics are produced by foaming ED-16 epoxy resin with refrigerant-113, curing it with the help of cationic polymerization catalysts.

It should be noted that the overwhelming majority of domestic epoxy composites (including PTM) have an increased fire hazard (flammability, smoke forming ability and toxicity of pyrolysis and combustion products) [11,12,13]. The importance of reducing the fire hazard of polymer composite materials (PCMs) and fire protection of various building structures on their basis is evidenced by numerous reviews of scientific and technical literature published over the last 5–6 years and the topics which cover almost all the issues of the fire-safe application of PCMs [14,15,16,17,18,19,20]. In [21], the possibility of obtaining fire-resistant constructional materials by modifying epoxy-novolac foams with oxidized or thermally expandable graphite was shown. Article [22] demonstrates that the best combination of flammability-reducing fillers for the epoxy composite matrices consisted of 15 wt.% ammonium polyphosphate, 5 wt.% dipentaerythritol and 3 wt.% organofilized bentonite. The synergy of silicon and phosphorus in this composition provided the material of UL-94 flammability class V-0. Epoxy resin-modified polyisocyanurate (EP-PIR) foams were successfully prepared by the reaction of polymethylene polyphenylene isocyanate (PAPI) and diglycidyl ether of bisphenol-A (DGEBA) [23]. Thermogravimetric analysis coupled with infrared spectrometry (TGA-IR) was performed to investigate the thermostability and gaseous pyrolysis products of EP-PIR foams. The results indicated that the excellent thermostability of EP-PIR foams was due to the abundant highly thermostable isocyanurate ring and oxazolidone ring. The flame retardation of EP-PIR foams was also investigated. UL94 results revealed that foams with a [PAPI]/[DGEBA] ratio above 2.5 could reach V-0 classification. With increasing the stoichiometry of [PAPI]/[DGEBA], the limiting oxygen index values increased linearly from 24.5 to 30.0 vol%. The scanning electron microscopy images of burned foams illustrated that the flame-retardant mechanism was due to the excellent charring ability. Cone calorimeter tests showed that the peaks of the heat release rate were always as low as 266.5 kW/m^2^. Moreover, a highly thermostable isocyanurate ring played an important role in the suppression of smoke emission [23]. The authors [24] established that sodium bicarbonate at the studied concentrations (10 and 15 wt%) is not appropriate for use as a filler capable of improving the thermo-oxidative stability and reducing the flammability of epoxy polymers. The improvement in the thermal properties can be achieved by using the combination of boric acid and multi-walled carbon nanotubes as fillers. The thermo-oxidative destruction of the samples filled with boric acid passes more slowly and more evenly via the formation of B_2_O_3_ as a result of its decomposition.

The originality of the presented study is due to the fact that in it, the influence of phosphorus- and bromine-containing flame retardants on the flammability and thermal resistance of epoxy foams was studied for the first time.

## 2. Materials and Methods

Casting epoxy foams were obtained on the basis of the diane epoxy resin of the ED-20 brand (GOST R 56211-2014, LLC PKF KhimAvangard, Dzerzhinsk, Russia). As an amine hardener, a mixture of polyethylene polyamine of directed synthesis (polyaminoalkylimidazoline) of UP-0641D grade (TU 6-05-241-202-82, JSC ENPC EPITAL, Moscow, Russia) and polyethylene polyamine (PEPA, TU 2413-010-75678843-2012, JSC ENPC EPITAL, Moscow, Russia) in the ratio of 4:1 was used. Organosilicon hydrophilizing liquid of 136-41 grade (GOST 10834-76, Khimtekh-R LLC, Lyubertsy, Russia) was used as a chemical-foaming agent to produce polyepoxide foams, and an organosilicon compound of KEP-2A grade (TU 20.14.51-309-00209013-2020, State Research Centre of the Russian Federation JSC ‘GNIICHTEOS’, Moscow, Russia) was used as a surfactant. Phosphate plasticizers (tricresyl phosphate, diphenyl (2-ethylhexyl) phosphate, di(2-ethylhexyl)phenyl phosphate (TU 6-06-241-92, LLC Research and Production Kamskaya Chemical Company, Perm, Russia), diphenylisopropyl phosphate (TU 6-05-211-1211-80, LLC Research and Production Kamskaya Chemical Company, Russia) and trichloropropyl phosphate (TU 2493-513-05763441-2007, LLC Research and Production Kamskaya Chemical Company, Russia), and chlorinated paraffin Parachlor-380 (TU 2493-005-1316440-92, LLC Research and Production Kamskaya Chemical Company, Russia) were used. Hexabromobromobenzene, tetrabromodiphenylpropane, tetrabromophthalic anhydride, decabromodiphenyloxide and N(2,4,6-tribromophenyl) maleinimide were used as additive organobromine flame retardants, and as reactive flame retardants, bromine-containing epoxy resins of UP-631 (TU 2225-652-11131395-2008, LLC Research and Production Kamskaya Chemical Company, Russia) and UP-645 (TU 6-05-241-40-82, LLC Research and Production Kamskaya Chemical Company, Russia) grades, containing 46.2 and 50.8% of bromine, respectively, as well as the epoxidized wastes of tetrabromodiphenylpropane production (EOTBDP), containing 44.3% of bromine and 10.5% of epoxy groups, were used. For comparison, phosphorus-containing oligoether methacrylate (2-phosphonoxyethyl methacrylate) containing 14.3% phosphorus was used. When preparing epoxy foam samples, the standard ratio of components was used; curing was carried out at a temperature of 25 °C.

**Figure 1:**
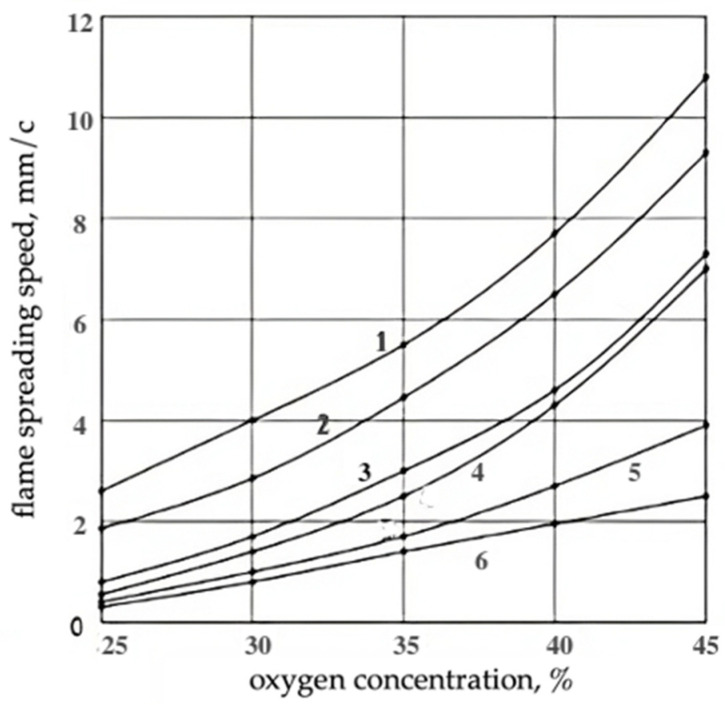
Dependence of flame propagation speed on the horizontal surface of filling epoxy foams of different densities on the concentration of oxygen in the oxidant stream and apparent density of RPE: 1—90.3 kg/m^3^, 2—115.2 kg/m^3^, 3—164.4 kg/m^3^, 4—172.1 kg/m^3^, 5—250.6 kg/m^3^, 6—315.6 kg/m^3^.

**Figure 2:**
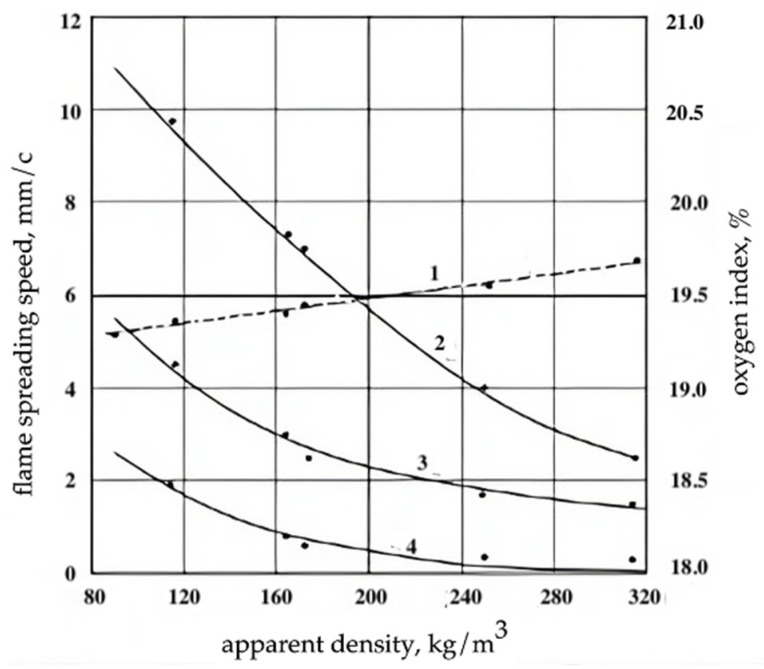
Dependence of oxygen index (1) and flame spreading speed on horizontal surface (2, 3, 4) of filling epoxy foams on their apparent density and oxygen concentration in oxidant flow: 2—45%; 3—40%, 4—35%.

**Figure 3:**
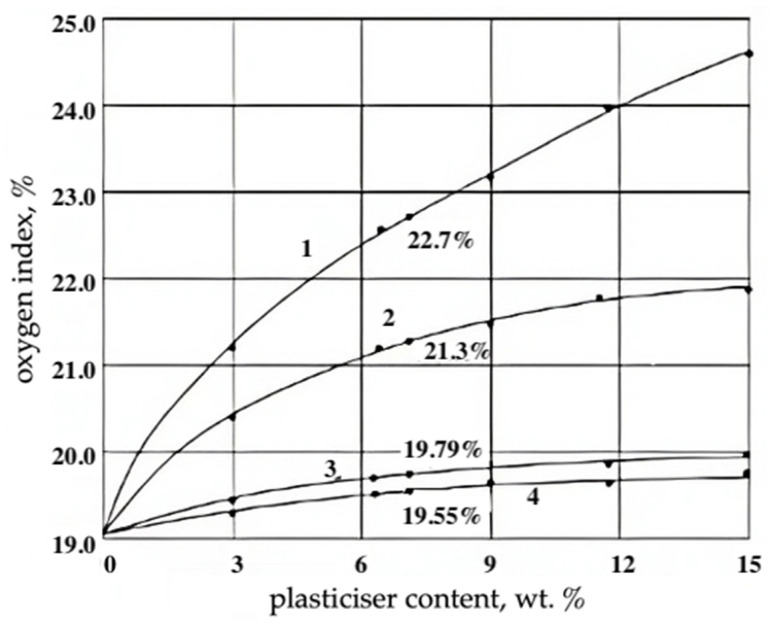
Dependence of oxygen index of filling epoxy foams on the content of plasticizers: 1—Parachlor-380 chlorparaffin; 2—trichloropropyl phosphate; 3—diphenyl(2-ethylhexyl)phosphate; 4—di(2-ethylhexyl)phenylphosphate.

**Figure 4:**
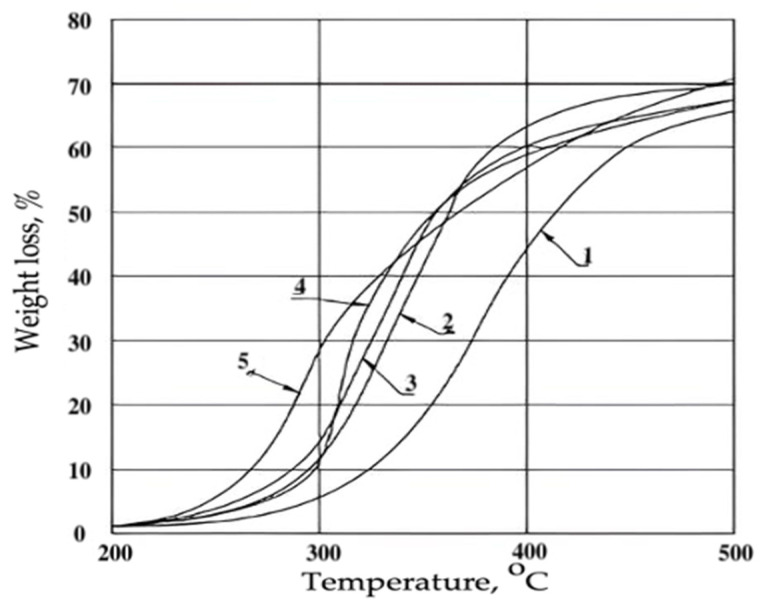
Thermogravimetric curves of the filling epoxy foams of reduced flammability: 1—without flame retardant; 2—N(2,4,6-tribromophenyl)maleinimide; 3—decabromodiphenyloxide; 4—tetrabromodiphenylolpropane; 5—chlorinated paraffin of the Parachlor-380 brand.

**Figure 5:**
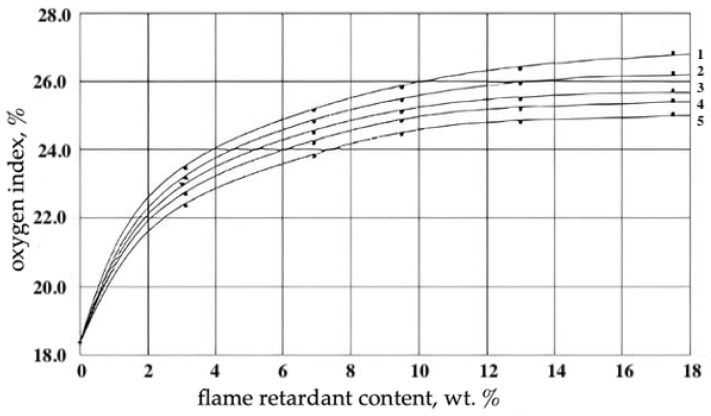
Dependence of the oxygen index of filling epoxy foams on the content of additive organobrominated flame retardants: 1—N(2,4,6,-tribromophenyl)maleinimide; 2—tetrabromodiphenyllolpropane; 3—hexabromobenzene; 4—decabromodiphenyloxide; 5—tetrabromphthalic anhydride.

**Figure 6:**
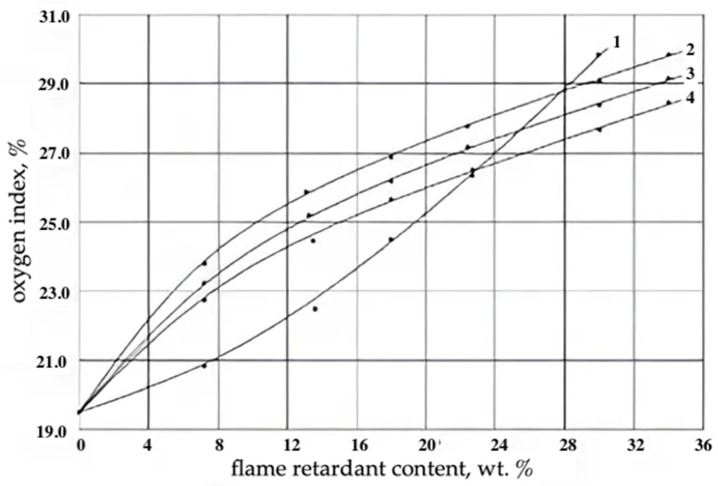
Dependence of the oxygen index of epoxy filling foams on the content of reactive flame retardants: 1—2-phosphonoxyethylmethacrylate; 2—epoxy resin of UP-645 grade; 3—epoxy resin of UP-631 grade; 4—epoxidized wastes of tetrabromodiphenylpropane production.

Technological parameters of ignition and curing of polyepoxide foams were determined according to generally accepted methods, and their physical and mechanical parameters were determined in accordance with the requirements of current GOSTs. In the present work, more than 100 epoxy foam compositions were investigated, including 86 compositions containing different concentrations of plasticizers, as well as additive and reactive organobromine and organophosphorus flame retardants (see Table 1 and Table 2, and Figure 1, Figure 2, Figure 3, Figure 4, Figure 5 and Figure 6). Apparent density was determined by GOST 409-2017 (ISO 845:2006), according to which the linear dimensions of the samples were measured, and their volume was calculated and weighed. Compressive strength was determined by GOST 23206-2017 (ISO 844:2014): the test specimen was placed between the two base plates of the testing machine so that the applied force coincided with the direction of foaming. The test specimen was compressed at a rate as close as possible to (10 ± 1) % of its original thickness per minute. The specimen was compressed and load–displacement curves were recorded. Water absorption was determined by GOST 20869-2017 (ISO 2896:2001). Thermal conductivity was determined by GOST 7076-99 by measuring thermal resistance and effective thermal conductivity using an instrument equipped with a heat meter and a hot guard instrument. Oxygen index (OI), ignition temperatures (T_I_) and self-ignition temperatures (T_SI_) were determined according to GOST 12.1.044-89 (ISO 4589-84). The limiting oxygen concentration (C_LIM_) and flame propagation velocity (V_P_) on a horizontal surface at an oxygen concentration in the oxidizer stream from 20 to 75% were investigated according to the method of work [25], and the critical density of ignition heat flux (*q_cr_*) was studied according to the method of work [26]. The thermal characteristics of the investigated PEFs were determined using the DuPont-9900 thermoanalytical complex (TA Instruments/Intertech Corp., New Castle, DE, USA) when the samples were heated in air at a rate of 10 and 20 °C/min, taking into account the requirement of GOST 53293-2009. Criteria for the thermal stability of epoxy foams were the temperatures of the start of intensive decomposition (T_sd_) and the maximum rate of decomposition (T_max_).

## 3. Results and Discussion

As a result of the performed experimental studies, it was found that the heat resistance and flammability of the studied epoxy filler foams practically does not depend on the chemical nature of the amine hardener: T_sd_ was 230–240 °C, T_max_ was observed at a temperature of 330–340 °C and OI was 19.2–19.7%. With an increase in the heating rate of foam samples from 10 to 20 °C/min, the T_sd_ of polyepoxides increased slightly: from 240 to 250 °C. The flame propagation velocity along the horizontal surface of the PEF depended on the oxygen concentration in the oxidizer stream (Figure 1) and the density of the foam (Figure 2).

The concentration of carbon oxides in the pyrolysis products of epoxy foams at 450–750 °C depended slightly on the content of the curing agent in the initial composition and was as follows:
T, °C[CO/CO_2_], volume, %450-0.25–0.26/0.42–0.46600-0.75–1.00/8.00–12.20750-0.20–0.25/16.50–16.90

At the same time, the maximum CO yield was realized at the temperature of 600 °C. The above data indicate the increased fire hazard of filling PEFs, which significantly limits their application in the construction industry.

Organic esters of orthophosphoric acid and liquid chlorinated paraffins were used to improve the technological properties and performance of epoxy polymers [10,27]. Chlorinated paraffins are weakly polar substances, having a polar group (chlorine) and a nonpolar hydrocarbon skeleton, which allows them to combine well with ED-20 resin to form a homogeneous composition. As a result of the conducted research, it was found that phosphate plasticizers and chlorinated paraffins (Parachlor-380) insignificantly affect the technological properties of casting RPE (start time and duration of foaming were, respectively, 160–180 s and 14–18 min), and the temperature in the block did not exceed 90 °C. However, plasticizers reduced the viscosity of the initial composition, which worsened the foam microstructure.

The flammability of the studied RPE was practically independent of the chemical structure of phosphate plasticizers: the oxygen index of foams at the content of 6.9 wt.% of orthophosphoric acid esters was 19.5–21.2% (Table 1), and the critical ignition heat flux density *q_cr_* = 18.3–19.3 kW/m^2^. The effect of the content of these plasticizers on the flammability of RPE is shown in Figure 3. The data in Table 1 and Figure 3 confirm the conclusions of the works [28,29] that orthophosphoric acid esters are ineffective flame retardants for PCM. Of the studied organic esters of orthophosphoric acid, diphenyl(2-ethylhexyl) phosphate had a high plasticizing effect, which had good compatibility with the epoxy oligomer. Parachlor-380 chlorinated paraffin had a higher flame extinguishing efficiency (Figure 3). The mechanism of inhibition of combustion of RPE in its presence was due to the phlegmatization of the flame, and reduction in the concentration of active radicals ($H^{\cdot }$, ${OH}^{\cdot }$ and $O^{\cdot }$) due to their interaction with the product of dehydrochlorination of chlorparaffin—HCI. In this case, the CI increased to 24.6 (Figure 3) and $q_{cr}$ was 22.3–23.5 kW/m^2^.

Table 1 shows that when 6.9 wt.% of orthophosphoric acid esters was introduced into the initial polymer composition, the concentration of phosphorus ([p]) in the foams was 0.54–0.66% and the oxygen index was 19.5–21.2%. Meanwhile, it was not possible to establish the influence of [p] in foams on the above indices for different types of phosphate plasticizers. This is due to the fact that the effectiveness of the flame-extinguishing action of these plasticizers is determined primarily by their chemical structure.

In addition, Parachlor-380 increases the deformation characteristics and reduces water absorption of RPE. In terms of water resistance, foams plasticized with chlorparaffin of Parachlor-380 grade were superior to RPE modified with other compounds. It should be noted that Parachlor-380 reduces the thermal stability of RPE (Figure 4) and practically does not affect the yield of carbon oxides during the decomposition of foams in the temperature range of 450–750 °C. With the increase in pyrolysis temperature, there was a regular increase in CO and CO_2_ concentration. When the RPE contained 11.75 wt.%. Parachlor-308, the concentration of CO and CO_2_ increased from 0.025 and 0.4 vol% at 450 °C to 2.25 and 15.5 vol% at 750 °C, respectively. The maximum yield of CO_2_, as with the original polyepoxy foam, was observed at 600 °C. The epoxy foams plasticized with Parachlor-380 were among the materials with a high smoke production capacity D_m_ > 500 m^2^/kg.

To reduce the flammability of foams based on reactive oligomers, nitrogen-containing organic compounds are widely used, which decompose when heated with the release of nitrogen, causing phlegmatization of the flame (for example, ChHZ-18 porophore). However, in epoxy foams, porophore CHHZ-18 has a low efficiency. At its content of up to 11 wt.%, the CI of foams increased from 19.5 to 23.1%, and the apparent density of PEFs increased up to 192 kg/m^3^ and, accordingly, the strength of polyepoxy foams increased (breaking stress in compression increased from 1. 2 to 2.24 MPa). A similar effect was achieved by introducing into the initial composition 10 wt.% of microencapsulated carbon tetrachloride or hladon II 4B-2 with particle diameter ˂ 160 μ (microcapsule shell: polyvinyl alcohol). The CI of foams was 22.7–23.5% at a foam density of 215–235 kg/m^3^. The reduction in flammability of PEFs in the presence of microencapsulated flame retardants was due to the effect of polymer dispersion and flame knockdown as a result of microcapsule rupture, phlegmatization and inhibition of flame chain reactions by the decomposition products of hladon or CCl_4_.

Organobromine flame retardants of additive type have higher flame retardant efficiency (Table 2). The mechanism of inhibition of PCM combustion in their presence is considered in works [30]. It follows from the data of Table 2 that at the content of organobrominated anitipyrenes 6.25% wt.%, the CI of epoxy foams increased up to 25.1–25.3%; at the same time, the thermo-oxidative stability of PCMs decreased slightly and their T_sv_ increased up to 480–490 °C. The effect of the content of organobromine flame retardants on the flammability of castable EPS is shown in Figure 5. The data of Table 2 and Figure 5 show that the chemical structure of additive organobromine flame retardants insignificantly affects their effectiveness.

To obtain moderately flammable filling foams based on reactive oligomers, according to [31], the CI should exceed 26%. It follows from Figure 5 that for the studied PEFs that the indicated effect is achieved at the content of additive organobromine flame retardants (N(2,4,6-tribromophenyl)maleinimide and tetrabromodiphenylolpropane) of more than 12.0 wt.%, i.e., when the concentration of bromine in the polymer matrix exceeds 7.3 wt.%. It should be noted that additive flame retardants in the process of the long-term operation of fire-protected heat-insulating products are prone to migration from the polymer matrix, which leads to an increase in the flammability of foams and a decrease in their performance characteristics. Therefore, it is preferable to use reactive bromine- and phosphorus-containing flame retardants. The influence of the content of reactive bromine- and phosphorus-containing flame retardants on the flammability of cast epoxy foams is shown in Figure 6.

Table 2 and Figure 5 show that 25 samples of epoxy foams containing different concentrations of additive organobromine flame retardants were investigated. Figure 6 shows the effect of the concentration of four kinds of reactive organophosphorus and organobromine flame retardants. The presented data indicate that the effectiveness of organobromine flame retardants depends primarily on their chemical structure, which has a significant effect not only on the flammability of epoxy foams, but also the density of foams and, consequently, the performance characteristics of foams. The experimental data obtained (Figure 6 and Table 2) show that bromine-containing compositions suppress the reactions occurring in the gas phase in the flame, thereby reducing the combustion efficiency. This is probably due to the fact that combustion is a chain radical process, and the introduction of halogen atoms contributes to a strong reduction in the energy of the radicals formed and accelerates their neutralization. Organophosphorus additives act differently: at elevated temperatures and combustion, they form a solid layer (coke) on the polymer surface with the formation of cross-linked structures. The solid coke residue was formed due to the formation of polyphosphoric acid and the carbonization reaction with the release of water, which also helps reduce flammability by diluting the gases formed. From the data in Figure 6, it can be seen that the use of organophosphorus additive in the amount of 30% by weight in the foam composition leads to a significant increase in the oxygen index (curve 1). This indicates the higher efficiency of organophosphorus flame retardants compared to other formulations. The course of curve 1 in Figure 6 is different from that of curves 2, 3 and 4. This confirms the different mechanisms of the flame retardant action described above for formulations of different chemical natures.

**Figure 7:**
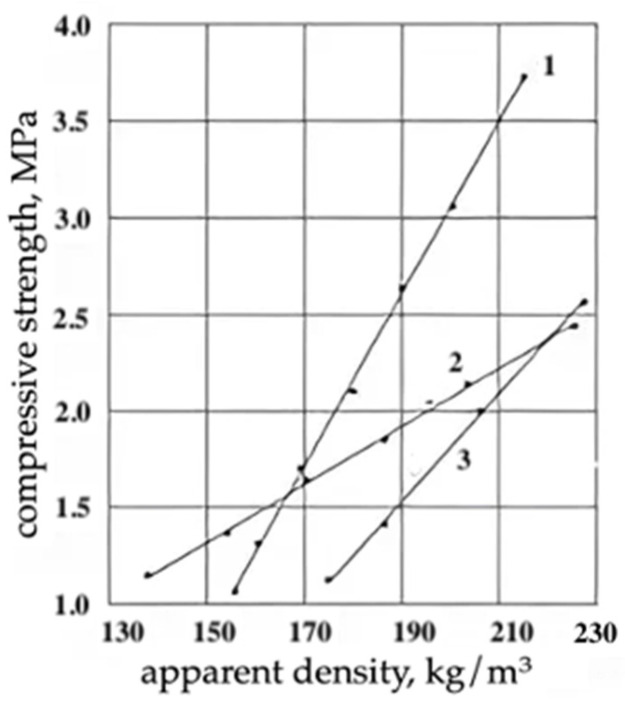
Dependence of compressive strength on the apparent density of filling epoxy foams: 1—initial EPS; 2—EPS containing phosphorus-containing oligoethermethacrylate as a fire retardant; 3—EPS containing epoxidized tetrabromodiphenylolpropane production waste as a fire retardant.

The use of epoxidized wastes of tetrabromodefinylolpropane production as flame retardants is a promising direction to reduce the flammability of epoxy foams for construction purposes. The oxygen index of PEFs at their content of more than 17 wt.% exceeded 27%. It should be noted that as the EOTBDP content increased, the density of PEFs increased and, consequently, the strength of epoxy foams increased. At the same time, in terms of operational characteristics, PEFs modified with epoxidized wastes from the production of tetrabromodiphenylpropane were inferior to other types of modified polyepoxide foams, but had a significantly lower cost. A positive effect was also achieved when phosphorus-containing oligoether methacrylate was used (Figure 6). The main performance properties, flammability and heat resistance of filling epoxy foams of reduced flammability are given in Table 3, and the dependence of the compressive failure stress on the apparent density of the developed PEFs for construction purposes is shown in Figure 7.

## 4. Conclusions

As a result of the conducted experimental studies, the influence of the chemical nature and content of phosphate plasticizers, and chlorinated paraffin of Parachlor-380, additive bromorganic and microencapsulated flame retardants, bromo- and phosphorus-containing reactive compounds on physical and mechanical properties, heat resistance and flammability of PEFs has been established.

The scientific novelty of the work consists in the following:

- The chemical nature of amine hardeners does not affect the thermal stability and flammability of epoxy foams.
- It has been established that phosphate plasticizers are ineffective flame retardants of foamed epoxy resin, and the chemical structure of additive organ bromic flame retardants insignificantly affects their efficiency.
- It is shown that microencapsulated flame retardants are inferior in flame-retardant efficiency to additive flame retardants. It has been found that effective flame retardants for casting polyepoxy foams are phosphorus-containing oligoether methacrylate and epoxidized waste from the production of tetrabromodiphenylpropane.
- As a result of the research developed moderately flammable filling epoxy foams (according to GOST 12.1.044-89, moderately flammable materials are those with a flue gas temperature not more than 235 °C, the degree of damage along the length of the tested sample not more than 85%, the degree of damage along the mass of the tested sample not more than 50%, and duration of independent combustion not more than 30 s) for construction purposes with high-performance characteristics.

## Figures and Tables

**Table 1:** The flammability, heat resistance, and physical and mechanical properties of plasticized (6.9 wt.%) filling epoxy foams.

Indicator	Grade of Phosphate Plasticizer				
	Trichloro-Propyl Phosphate	Diphenyl(2-ethyl-hexyl)-phosphate	Di(2-ethylhexyl)- phenyl Phosphate	Diphenyl Isopropylphenylphenyl Phosphate	Tricresyl Phosphate
Phosphorus concentration, wt.%, in:					
plasticiser	9.47	8.55	7.78	8.42	9.50
foam	0.65	0.59	0.54	0.58	0.66
Temperature of the beginning of intensive decomposition, °C	217	216	215	220	224
Oxygen index, %	21.2	19.7	19.5	19.8	19.7

**Table 2:** The flammability of filling epoxy foams containing organobromine flame retardants.

Indicator	Organobromic Flame Retardants				
	Hexabromobenzene	Tetrabromide Diphenylolpropane	Tetrabromophthalic Anhydride	N(2,4,6-tribromophenyl) Maleimide	Decabromodiphenyloxide
Bromine concentration, wt.%, in:					
- flame retardant;	85.5	58.5	67.5	58	82.5
- foam	5.90	4.04	4.66	4.00	5.69
Apparent density, kg/m^3^	157.2	149.3	144.5	139.7	140.1
Oxygen index, %	24.7	25.0	24.0	25.3	24.3
Temperature, °C:					
- beginning of intensive decomposition;	213	216	225	218	210
- maximum rate of decomposition;	315	321	325	317	335
- autoignition	490	480	470	460	490

Note—the content of organobromine flame retardants in PEFs is 6.25 wt.%.

**Table 3:** Physical–mechanical properties, flammability and thermal stability of filling epoxy foams.

Indicator	PET-20-Grade PPE	Initial Pouring Epoxy Foam Foam	Type of Reactive Flame Retardant	
			Phosphorus-Containing EMP	Wastes from Tetrabromodiphenylpropane Production
Apparent density, kg/m^3^	90–180	150–220	130–230	170–230
Destructive stress, MPa, at:				
- compression	0.25–2.20	1.07–3.70	1.15–2.65	1.12–2.55
- tensile	0.15–1.20	0.64–1.40	0.54–1.25	0.48–1.10
Water absorption in 24 h, % by volume	4.07–7.75	3.00–3.40	6.70–7.80	6.40–7.50
Thermal conductivity,	0.046–0.58	0.051–0.054	0.051–0.060	0.052–0.058
Oxygen index, %	18.7	19.5–19.8	27.2–28.5	28.4–29.2
Temperature, °C:				
- the start of intensive decomposition;	-	232–235	239–242	222–225
- max decomposition rate;	-	342–345	349–353	318–322
- ignition;	-	375	365	360
- self-ignition	-	460	490	480

## Data Availability

The original contributions presented in the study are included in the article; further inquiries can be directed to the corresponding author/s.
